# Global research status, hotspots and prospects of ferroptosis in glioma: a scientometric analysis

**DOI:** 10.3389/fncel.2025.1614710

**Published:** 2025-08-01

**Authors:** Jing Liu, Zefeng He, Zhansen An, Shiduo Li, Liang Liu, Yingzi Liu

**Affiliations:** ^1^Department of Neurosurgery, The Fourth Hospital of Hebei Medical University, Shijiazhuang, Hebei, China; ^2^Institute of Tumor, The Fourth Hospital of Hebei Medical University, Shijiazhuang, Hebei, China

**Keywords:** glioma, ferroptosis, bibliometrics, VOSviewer, CiteSpace

## Abstract

**Objective:**

The primary approach to the treatment of glioma involves surgical intervention, which is often complemented by radiotherapy, chemotherapy, and electric field therapy. Nevertheless, the prognosis for many patients remains poor. There exists an urgent necessity to identify novel replacement therapy strategies. A great breakthrough has been made in the study of ferroptosis in gliomas. The objective of this study is to conduct a systematic review of the current research status of ferroptosis in gliomas utilizing bibliometric analysis.

**Method:**

Publications related to glioma and ferroptosis from 2014 to 2025 were retrieved from the Science Core Collection (WoSCC) database. A bibliometric analysis was conducted using VOSviewer, CiteSpace, and the R package.

**Results:**

A total of 440 publications from 38 countries, with China leading the way, were included in the analysis. The number of publications related to ferroptosis in gliomas has been steadily increasing each year. The primary research institutions contributing to this field include Central South University, Nanjing Medical University, Shandong University, and Southern Medical University. Frontiers in Oncology is the leading journal for ferroptosis research in gliomas, while Cell is the most frequently cited journal in this field. These publications were authored by 2,921 individuals. Among them, Lu Shan, Wang Xuanzhong, Chen Qianxue, Sun Qian, and Xu Yang contributed the highest number of articles, while Dixon, SJ was the most frequently co-cited author. Studying the mechanisms, targets, and immunotherapy of ferroptosis in gliomas are major topics in this field. “Oxidative stress,” “gpx4,” and “autophagy” are popular keywords in recent years. In addition, “chemotherapy” and “miRNA” are emerging topics in this field that are closely related to this study and warrant greater attention.

**Conclusion:**

This is the first bibliometric analysis of the relationship between ferroptosis and glioma conducted over the past 11 years. The analyzed studies elucidate the regulatory mechanisms of ferroptosis and its implications in cancer cells, identify emerging research hotspots and Frontiers in recent years, and offer valuable references for scholars investigating ferroptosis in glioma, thereby facilitating the exploration of novel alternative treatment options.

## 1 Introduction

Glioma represents the most prevalent type of tumor found within the central nervous system (Bray et al., 2024; Ge et al., 2021). Despite the current treatments available for glioma, the prognosis for most patients remains poor, with an average life expectancy of approximately five years following diagnosis (Puliyappadamba et al., 2014). Glioma continues to present significant challenges to oncology research due to its high invasiveness and resistance to treatment. Recent studies have demonstrated that gliomas exhibit significant heterogeneity, characterized by a diverse array of molecular subtypes. These subtypes frequently present with a range of clinical manifestations and exhibit varying responses to different anti-tumor therapies (Comba et al., 2021). This brings a very severe test for the effective treatment of glioma. At present, the treatment of glioma primarily relies on conventional methods, including surgery, radiotherapy, and chemotherapy. However, these conventional methods exhibit limited inhibitory effects on glioma and fail to meet clinical needs. It is important to note that, despite the numerous challenges associated with glioma treatment from various perspectives, inducing the death of glioma cells remains an effective therapeutic approach (Fang X. et al., 2023; Fang Y. et al., 2023). Therefore, new therapeutic modalities and targets with high sensitivity and specificity still need to be identified (Peng et al., 2023).

Ferroptosis is a form of programmed cell death that results from the accumulation of lipid hydroperoxides (Jiang et al., 2021). Ferroptosis is an iron-dependent form of regulated cell death influenced by various factors. The induction of ferroptosis in tumor cells has emerged as a novel approach in cancer therapy. Research has demonstrated that glioma cells exhibit ferroptosis in response to a range of stimuli (Upadhyayula et al., 2023) and to be resistant to ferroptosis by some underlying mechanism (Luo et al., 2022). The dysregulation of ferroptosis is a key link affecting the proliferation and invasion of glioma, and this complex regulatory mechanism also provides new potential targets for the treatment of glioma.

Bibliometrics aims to analyze the structure and direction of research in relevant scientific fields within a specific time period (Chen and Song, 2019; Synnestvedt et al., 2005; van Eck and Waltman, 2010). Bibliometrics is an emerging method of digital information statistics that enables both quantitative and qualitative analysis of literature in a systematic manner, revealing the quantitative characteristics of scholarly works. This specialized analytical approach allows for the digital evaluation of the distribution, connections, and patterns within a particular field of study (Li et al., 2021). On the other hand, it can also illustrate the primary content and evolutionary process of the research field through the construction of citation networks, co-author collaborations, and keyword co-occurrences (Brika et al., 2021; Cruz-Cárdenas et al., 2021; Donthu et al., 2021; Sidhu et al., 2020). Nowadays, bibliometrics plays a significant role in the field of medical research, thanks to the flexibility of development tools like RStudio and their powerful statistical analysis capabilities. Biblioshiny (Bibliometrix, Naples, Italy) can be utilized as a web-based tool to integrate literature into RStudio. Through its user-friendly interface, we can utilize straightforward programming languages for bibliometric analysis of the literature. This established the foundation for numerous literary analyses, including citation analysis and thematic evolution (Racine, 2012; Savita and Verma, 2020; Thomas et al., 2023). VOSviewer and CiteSpace are two analytical tools commonly used in bibliometrics. They are primarily used to construct and visualize the network of relationships in bibliometrics. VOSviewer can map and cluster keywords, journals, and authors within the literature, as well as construct interaction networks. CiteSpace can cluster the topics and keywords of the literature and visualize their evolutionary processes over time. These intuitive visualizations can help researchers understand and interpret complex bibliometric data more effectively. In addition, these tools can facilitate an in-depth exploration of the published literature, thereby revealing development trends and identifying major contributors in the research field (Agbo et al., 2021).

In the field of biomedicine, a substantial number of bibliometric studies have been undertaken. However, a bibliometric analysis specifically addressing ferroptosis in glioma has not yet been performed. This study presents a bibliometric analysis of publications related to ferroptosis in glioma over the past 11 years. The primary objective of this analysis is to identify recent research achievements and systematically outline the current body of literature in this area. Additionally, it aims to forecast future research trajectories and developmental opportunities concerning ferroptosis in glioma.

## 2 Materials and methods

### 2.1 Search strategy

**FIGURE 1:**
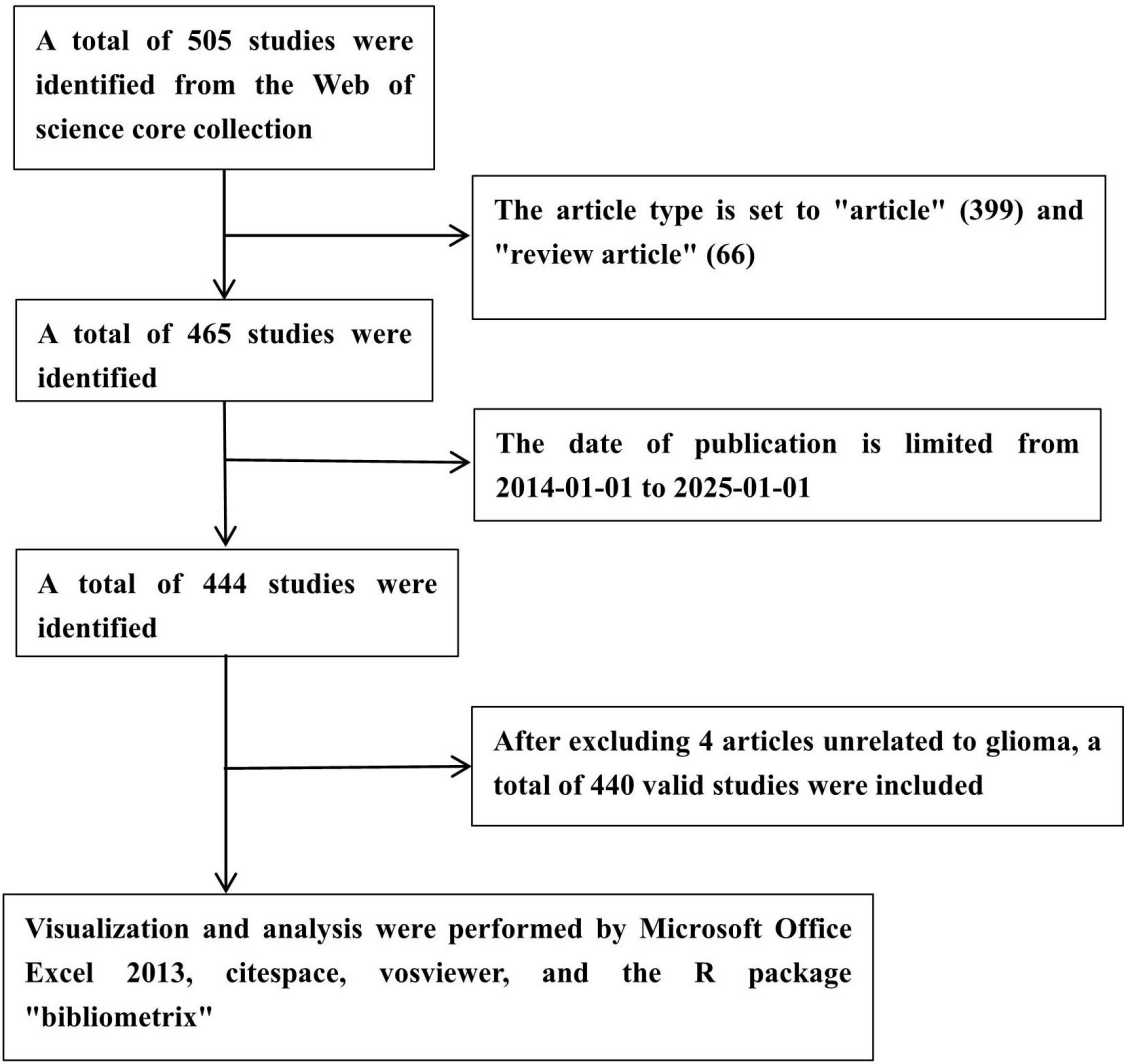
Literature screening process.

On 1 January 2025, we conducted a literature search using the Web of Science Core Collection (WoSCC) database.^1^ The search formula was TS = (“glioma*” OR “glioblastoma*” OR “GBM” OR “astrocytoma*” OR “oligodendroglioma*” OR “ependymoma*”) AND TS = (“ferroptosis” OR “iron death” OR “iron-dependent cell death” OR “iron mediated cell death” OR “lipid peroxidation-induced cell death”). By using this search formula and limiting the language to English, we obtained 505 relevant documents from WoSCC. Then we limited the literature types to “Review” and “Article,” and obtained 465 literatures through the initial screening. By further screening, we limited the publication dates to from 1 January 2014 to 1 January 2025, and as a result, 444 documents were obtained. Finally, we excluded 4 literatures unrelated to glioma and included a total of 440 literatures for bibliometric analysis. The data screening process is shown in Figure 1.

### 2.2 Data analysis

VOSviewer is a bibliometric analysis software that can analyze multiple pieces of literature and construct visual network maps. This study primarily conducted the following analyses: country and institution analysis, journal and co-cited journal analysis, author and co-cited author analysis, keyword co-occurrence analysis, and co-cited literature analysis. In the VOSviewer map, nodes represent various entities, including countries, regions, institutions, and researchers. The size of each node corresponds to the quantity of these entities, while the color of the node signifies their classification. Additionally, the thickness of the lines connecting the nodes illustrates the extent of collaboration or co-reference among the entities. When utilizing VOSviewer for analysis, we aim to present a comprehensive overview of the countries, institutions, journals, references, and keywords that have significantly contributed to this research. To ensure clarity and completeness of the visualizations while avoiding overlap, we establish appropriate visualization thresholds for each analysis. This approach guarantees the accuracy and reliability of the research findings.

CiteSpace can be utilized for bibliometric visualization. By employing CiteSpace, we performed keyword clustering and created a co-occurrence timeline view of the keywords.

R package “bibliometrix”^2^ to analysis various countries the number of articles and the highly cited papers. In addition, we mapped keyword clouds, conducted a topic evolution analysis, and constructed a global network of publications related to glioma and ferroptosis. Microsoft Office Excel 2013 was used for quantitative analysis of publications.

## 3 Results

### 3.1 Quantitative analysis of publications

**FIGURE 2:**
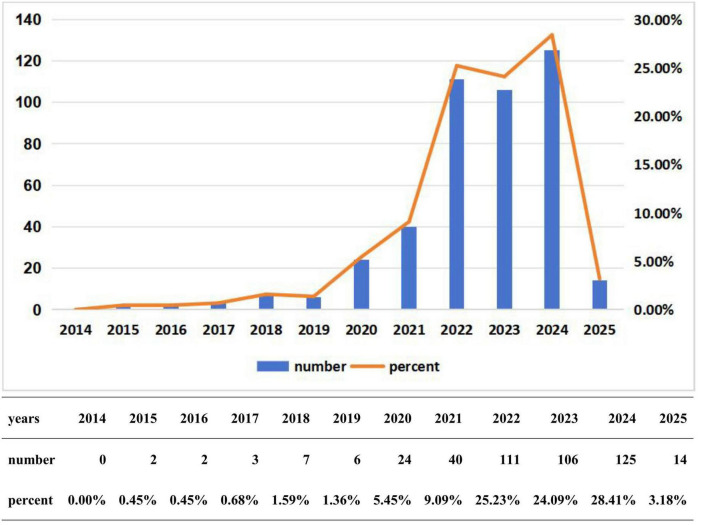
Annual output value of ferroptosis studied in gliomas.

After screening by article type, language, publication time and topic relevance, our study included a total of 440 meaningful documents. According to the number of publications (Figure 2), research on glioblastoma-related ferroptosis was virtually non-existent in 2014. The number of papers published from 2015 to 2019 was minimal, and growth during this period was slow, indicating that research was still in its early stages. However, after 2020, interest in this area began to increase significantly, particularly between 2022 and 2024, when the number of published papers experienced explosive growth. Over this period, the total number of published papers accounted for more than 50% of the total publications in the past 11 years, with an annual output exceeding 100 papers. As of now, there have been 14 published papers in 2025. Compared to previous years, research on ferroptosis in gliomas is expected to remain at a high level over the past four years.

### 3.2 Country and institutional analysis

**FIGURE 3:**
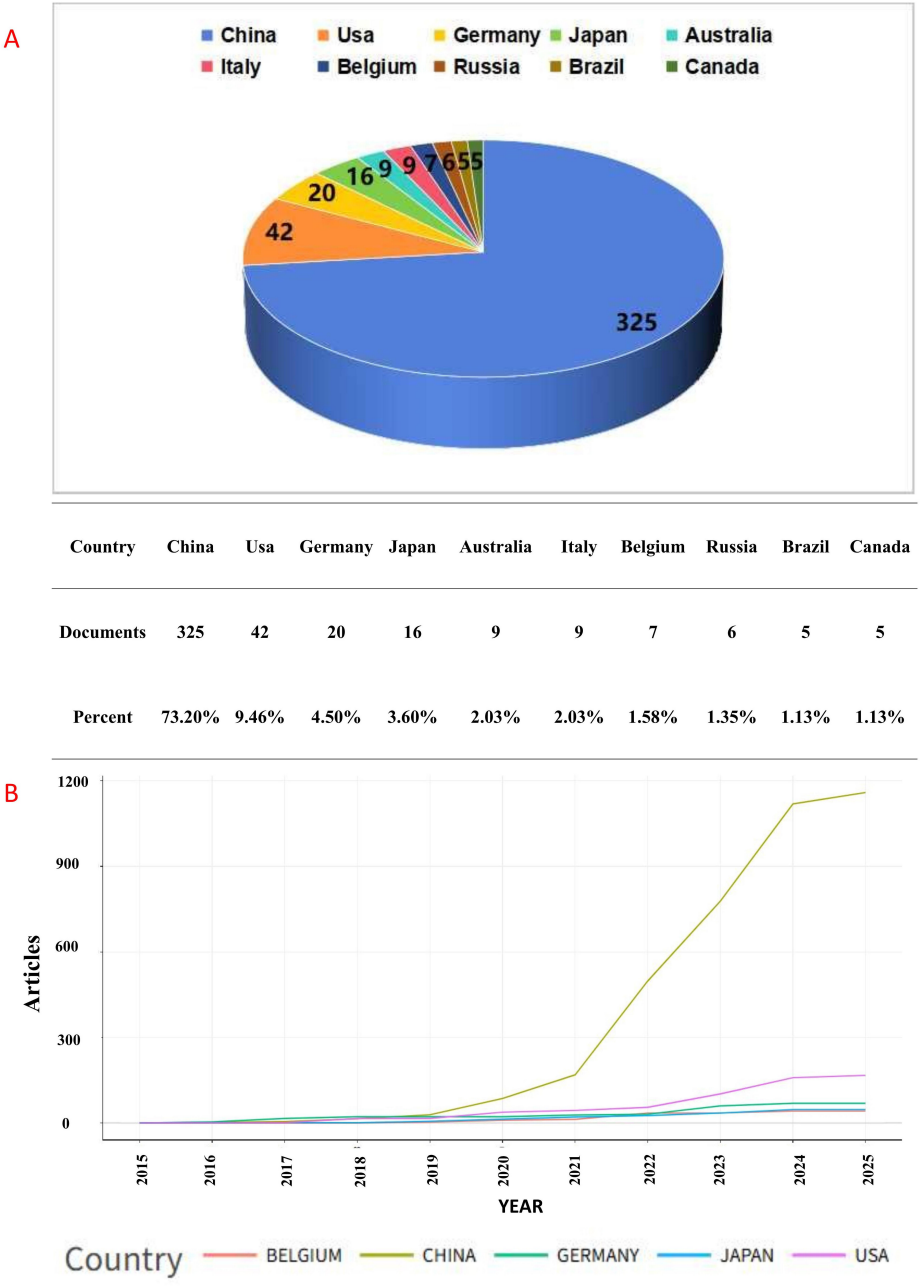
**(A)** pie chart of publications from the top 10 countries, and **(B)** chart of national publications over time.

These publications originate from 582 research institutions across 38 countries. The top 10 countries ranked by publication volume are illustrated in Figure 3A. Notably, China accounts for the highest percentage of publications at 73.20%, followed by the United States at 9.46%. Figure 3B illustrates the changes in the number of publications by country over time, revealing a consistent year-on-year increase in publication volume for each country. Notably, after 2020, China’s annual publication output has experienced explosive growth, placing it significantly ahead of other nations. Based on the analysis results in Figure 3, we find that Chinese scholars have invested a lot of energy in this field since 2020. A variety of practical research results have emerged, and they are now ahead of most countries in the world.

**FIGURE 4:**
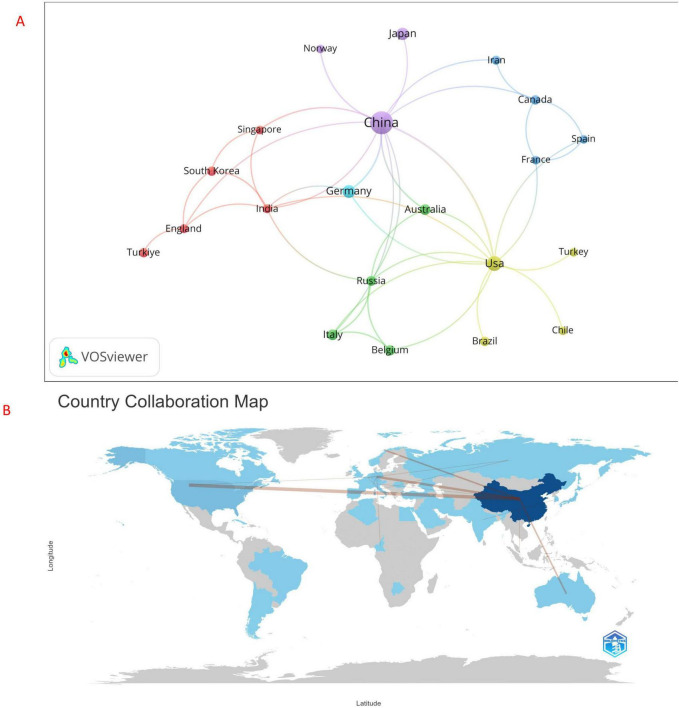
**(A)** national cooperation network, and **(B)** world map of national cooperation.

Therefore, to further analyze the connections between countries with significant research investments in this field, we filtered countries based on a minimum threshold of three publications and constructed an interaction network reflecting the number of publications and collaborative relationships among each country (see Figure 4). The results of Figure 4A indicate the existence of positive mutual cooperation among countries. China demonstrates close collaboration with the United States, Japan, Norway, Germany, Australia, Canada, Iran, and several other countries. Additionally, the United States actively cooperates with Australia, Russia, Belgium, France, Turkey, and others. Figure 4B illustrates a world map depicting cooperation among various countries. In this map, darker colors indicate a higher number of publications, while the connections between countries are represented by line segments. Thicker line segments signify greater levels of cooperation. Notably, China exhibits the darkest color, indicating the largest number of publications. The line segment between China and the United States is the thickest. The exchange programs carried out between the two are the most numerous.

**FIGURE 5:**
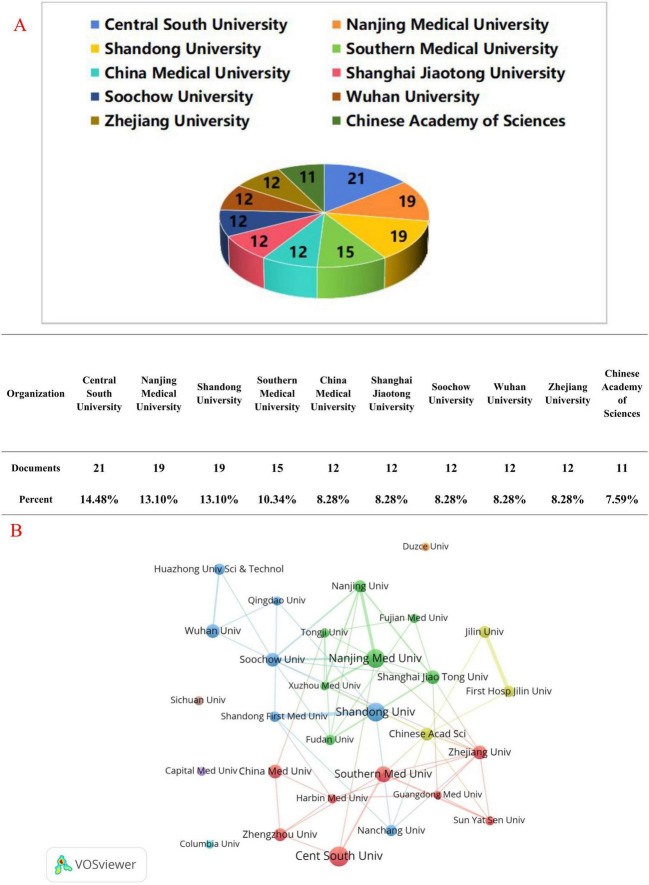
Publications **(A)** and network **(B)** of institutions conducting ferroptosis research in gliomas.

The top ten institutions ranked by publication volume are illustrated in Figure 5A, with nearly all of them located in China. The four institutions with the highest number of publications are Central South University (14.48%), Nanjing Medical University (13.10%), Shandong University (13.10%), and Southern Medical University (10.34%). We subsequently visualized institutions that had a minimum of six publications and constructed interaction networks based on the number of publications and collaborative relationships among each institution. As depicted in Figure 5B, Central South University demonstrates strong collaboration with Zhengzhou University, Harbin Medical University, Southern Medical University, and China Medical University. Additionally, Shandong University, Shandong First Medical University, Wuhan University, Qingdao University, and Huazhong University of Science and Technology, as well as Nanjing Medical University, Nanjing University, Fujian Medical University, Tongji University, and Xuzhou Medical University, also exhibit close cooperation. Jilin University, the First Hospital of Jilin University, and the Chinese Academy of Sciences maintain strong collaborative ties. Notably, we observed that Sichuan University, Capital Medical University, Columbia University, and Duzce University have published a significant number of papers but have not engaged in collaborations with other institutions.

### 3.3 Journals and co-cited journals

**FIGURE 6:**
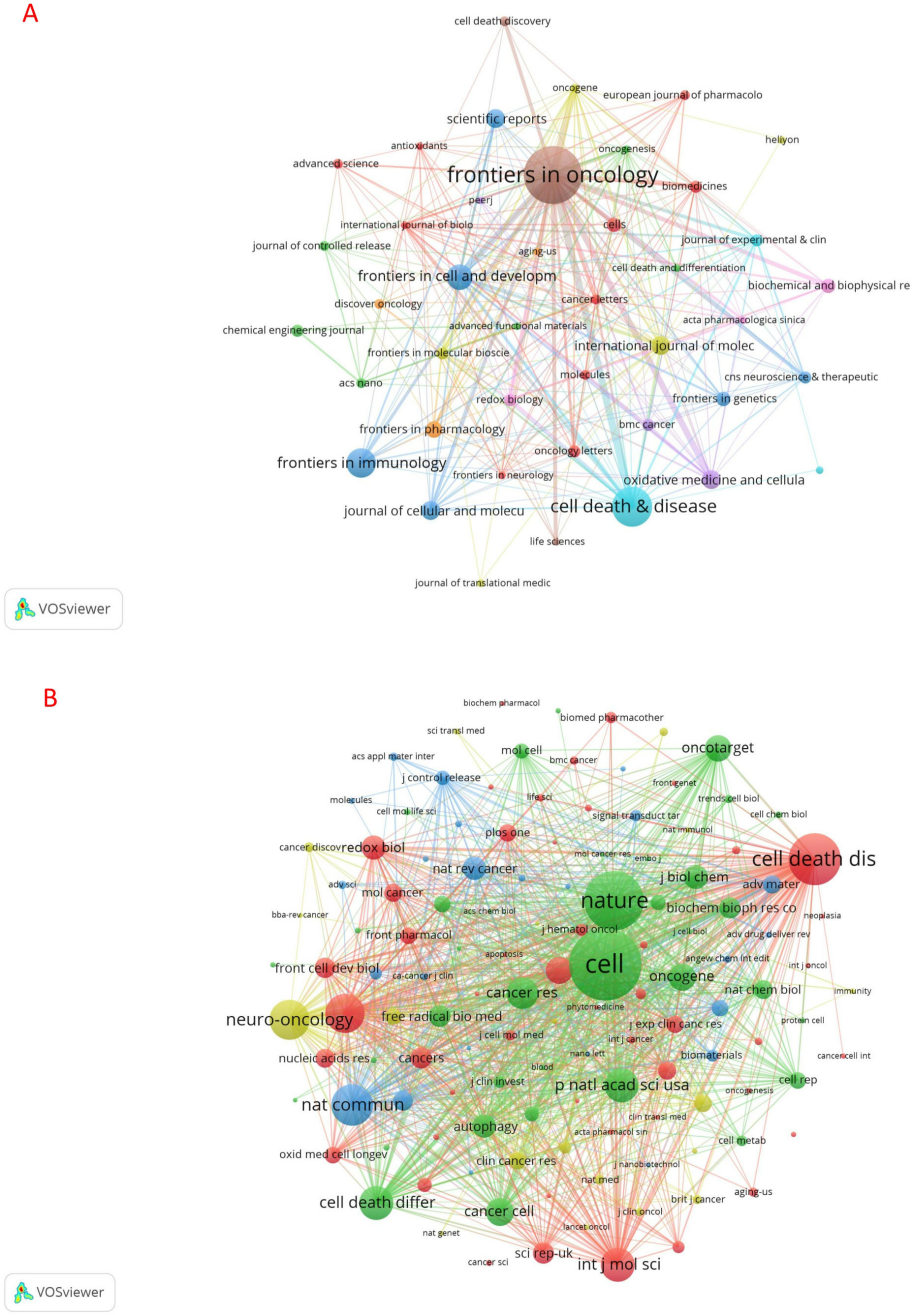
Visualization of journals **(A)** and co-cited journals **(B)** on ferroptosis studies in gliomas.

Publications related to ferroptosis in gliomas appeared in 200 journals, with the highest number of publications in Frontiers in Oncology (*n* = 27), followed by Cell Death & Disease (*n* = 18). Other notable journals include Frontiers in Immunology (*n* = 13) and Frontiers in Cell and Developmental Biology (*n* = 11). Using the 2024 impact factor as a standard, among the top ten journals by publication volume, Cell Death & Disease had the highest impact factor (IF = 8.1), followed by Frontiers in Immunology (IF = 5.7). We then selected 42 journals based on a minimum publication threshold of three and plotted the network of these journals (Figure 6A). The results presented in Figure 6A indicate that Frontiers in Oncology has highly active citation relationships with Cell Death & Disease, Scientific Reports, Cells, and others.

As shown in Table 1, the top 10 co-cited journals were each cited more than 300 times, with three journals receiving over 500 citations. Cell had the highest number of citations (co-citation = 784), followed by Nature (co-citation = 650) and Cell Death & Disease (co-citation = 570). Notably, Nature also had the highest impact factor (IF = 50.5), followed by Cell (IF = 45.6). The co-cited journals were filtered to include only those with a minimum of 50 co-citations, and a co-citation network map was created (see Figure 6B). Figure 6B illustrates the co-citation relationships between Nature and other journals, including Cell, Cell Death & Disease, Neuro-Oncology, and Cancer Research, among others.

**TABLE 1:** The top 10 journals and co-cited journals in glioma research.

Source	Documents	IF	Co-citation source	Citations	IF
Frontiers in Oncology	27	3.5	Cell	784	45.6
Cell Death & Disease	18	8.1	Nature	650	50.5
Frontiers in Immunology	13	5.7	Cell Death Discovery	570	6.1
Frontiers in Cell and Developmental Biology	11	4.6	Nature Communications	453	14.7
International Journal of Molecular Sciences	8	4.9	Neuro-oncology	438	16.4
Journal of Cellular and Molecular Medicine	8	4.3	Frontiers in Oncology	426	3.5
Oxidative Medicine and Cellular Longevity	8	0	International Journal of Molecular Sciences	371	4.9
Scientific Reports	8	3.8	Proceedings of the National Academy of the United States of America	368	9.4
Frontiers in Pharmacology	7	4.4	Cell Death and Differentiation	367	13.7
Biochemical and Biophysical Research Communications	6	2.5	Cancer Research	345	12.5

### 3.4 Authors and co-cited authors

**FIGURE 7:**
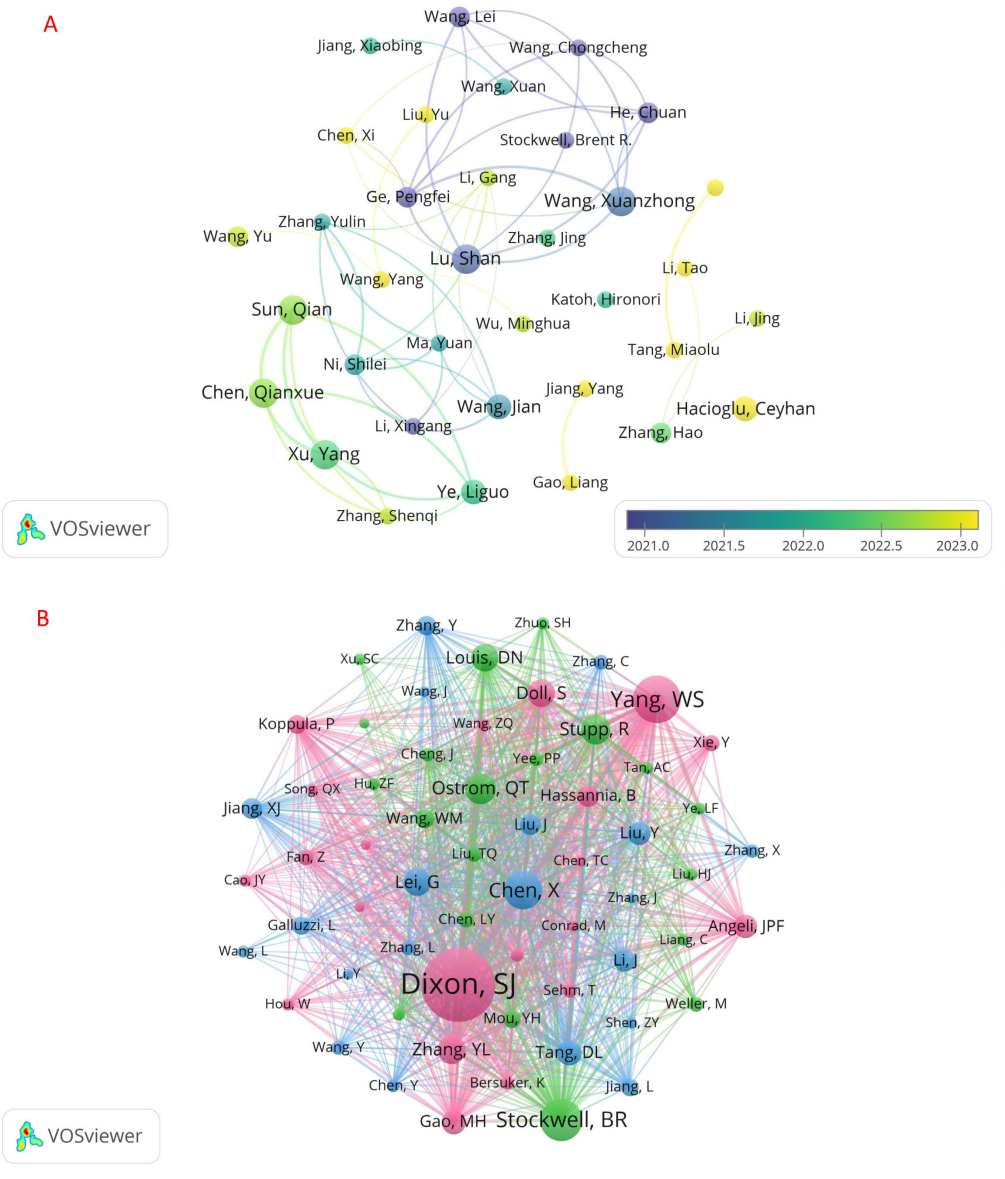
Studies of ferroptosis in gliomas by authors **(A)** and co-cited authors **(B)**.

A total of 2,921 authors were involved in the study of ferroptosis in gliomas. Among the top 10 authors (see Table 2), five authors published seven articles. We filtered and plotted the network based on a minimum publication threshold of four articles (see Figure 7A). As illustrated in Figure 7A, Lu Shan, Wang Xuanzhong, Chen Qianxue, Sun Qian, and Xu Yang published the most papers and had the largest nodes in the network graph. Lu Shan was the first to initiate research among the top 10 authors, and their node color was the darkest. Additionally, several authors collaborated closely, including Lu Shan and Ge Pengfei, Wang Xuanzhong and Wang Lei, as well as Chen Qianxue, Sun Qian, Xu Yang, and Zhang Shenqi.

**TABLE 2:** Top 10 authors and co-cited authors of ferroptosis studies in gliomas.

Author	Documents	Co-cited authors	Citations
Chen Qianxue	7	Dixon, SJ	166
Lu Shan	7	Yang, WS	106
Sun Qian	7	Stockwell, BR	86
Wang Xuanzhong	7	Chen, X	75
Xu Yang	7	Ostrom, QT	63
Hacioglu Ceyhan	6	Stupp, R	62
Wang Jian	6	Zhang, YL	55
Ye Liguo	6	Doll, S	55
Ge Pengfei	5	Lei, G	49
He Chuan	5	Louis, DN	46

Among the 14,585 co-cited authors, 2 authors were cited more than 100 times; the most frequently cited author was Dixon, SJ (*n* = 166), followed by Yang, WS (*n* = 106). The network of co-cited authors was plotted by filtering with a minimum co-citation count of 30 (Figure 7B). As shown in Figure 7B, there are also cooperative relationships among different co-cited authors, such as Dixon, SJ and Yang, WS, Zhang, YL, Gao, MH, etc.

### 3.5 References citation and co-cited references

**FIGURE 8:**
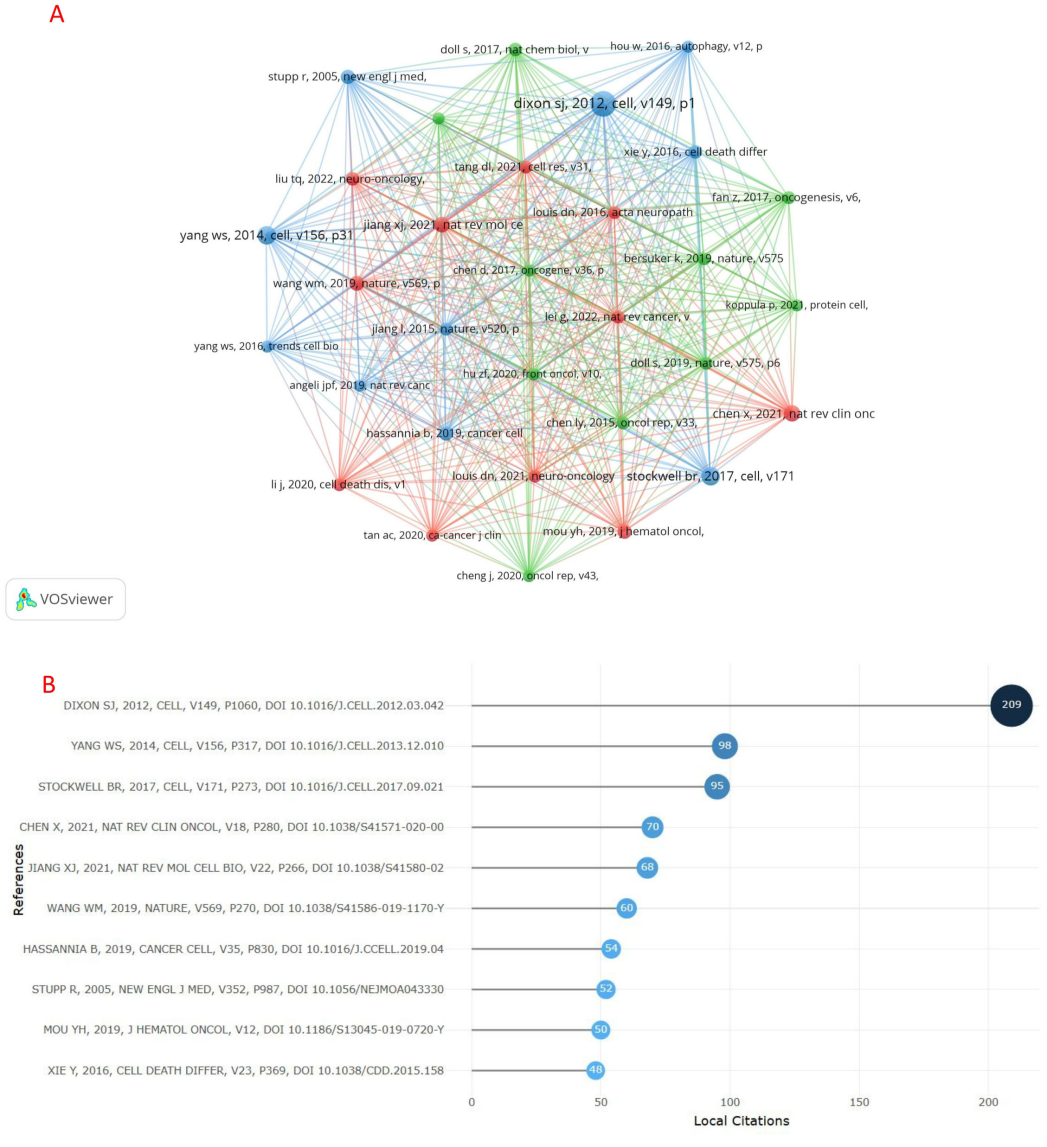
**(A)** co-cited references and top 10 references cited for studies **(B)** of ferroptosis in gliomas Visualization.

During the period from 2014 to 2025, there were 19,586 co-cited references on ferroptosis in gliomas. The minimum co-citation threshold for screening was set at 35, and the co-citation network graph was constructed using VOSviewer. The significant literature was categorized into three clusters, each represented by a distinct color (Figure 8A). As illustrated in Figure 8A, “DIXON SJ, 2012, Cell” and “YANG WS, 2014, Cell” and “HOU W,2016,autophagy,v12,p” and “XIE Y, 2016,” There is a very active co-citation relationship between “Cell Death & Differentiation” and others. The R package bibliometrix was utilized to identify the top 10 cited references (Figure 8B), all of which had been cited more than 40 times, with the most cited reference exceeding 200 citations. As shown in Figure 8B, cited the most were “DIXON SI, 2012, the CELL, V149 P1060, DOI 10.1016/1. CELL. 2012.03.042.” The title of the paper is “Ferroptosis: an iron-dependent form of non-apoptotic cell death.” Cited the second is “YANG WS, 2014, the CELL, V156 P317, DOI 10.1016/1. CELL. 2013.12.010.” The title of the paper is “Regulation of ferroptotic cancer cell death by GPX4.”

### 3.6 Research hotspots and Frontiers

**FIGURE 9:**
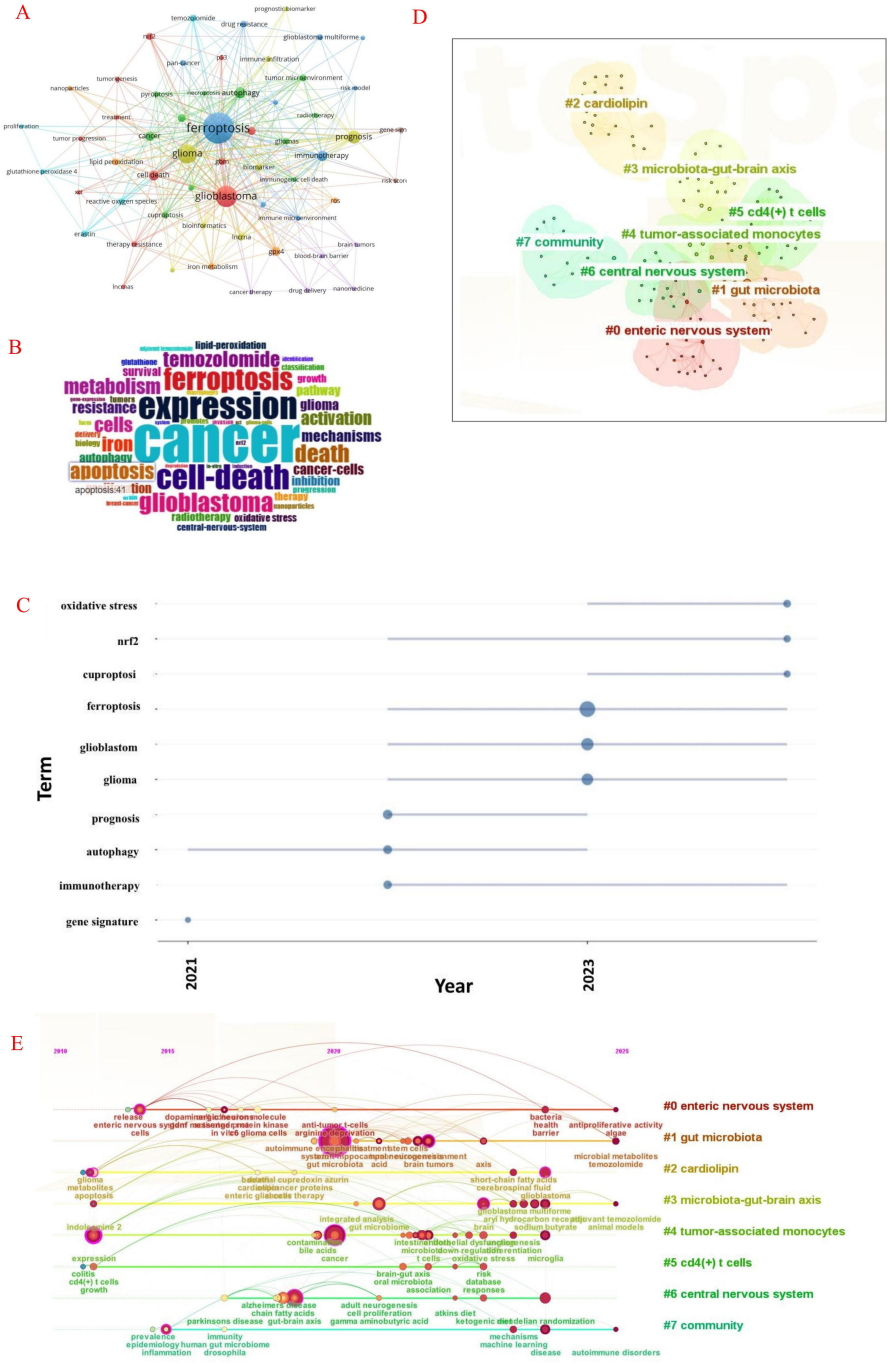
Keyword network diagram constructed by VOSviewer **(A)**, keyword cloud diagram constructed by R package bibliometrix **(B)** and trend keyword diagram **(C)**, keyword cluster analysis visualized by CiteSpace **(D)**, keyword timeline view **(E)**.

Through the analysis of keywords, we can swiftly identify the current research hotspots and focal points in this area. Table 3 presents the top 20 keywords related to ferroptosis in gliomas. Among these keywords, “prognosis,” “autophagy,” “cell death,” “apoptosis,” “gpx4,” “immunotherapy” appeared more than 20 times, and “oxidative stress,” “incRNA,” “biomarker,” “lipid peroxidation,” “NFR2,” “temozolomide treatment,” “tumor microenvironment” appeared more than 10 times. These keywords represent the primary directions and research trends of ferroptosis in glioma. We filtered keywords that appeared a minimum of four times and used VOSviewer to plot the interaction network of these keywords (Figure 9A). The larger the nodes, the more frequently the keywords appear; the thicker the lines connecting the nodes, the stronger the interaction between the keywords. This visualization not only highlights the frequency of keywords in the literature but also reveals the relationships among disciplines related to the research topic. The word cloud (Figure 9B) illustrates the most common keywords and terms, with their frequency represented by font size. We found that “cancer” was the most common term, followed by “expression,” “cell death,” “ferroptosis,” “glioblastoma,” “temozolomide,” “iron,” “cell” and “metabolism.” As illustrated in Figure 9C, between 2021 and 2023, researchers have been actively investigating the impact of ferroptosis on glioma survival, identifying target genes, and exploring its potential applications in immunotherapy. “Immunotherapy,” “prognosis,” “glioma,” “glioblastoma,” “gene signature,” “ferroptosis,” “apoptosis,” and “autophagy” were the main keywords in this period. After 2023, researchers will continue the previous studies and continue to study the role of ferroptosis in the regulation of “oxidative stress,” “NFR2,” and “copper death” in gliomas. Keyword clustering reveals the foundational aspects of a specific study, which aids in our classification efforts. CiteSpace was utilized to cluster keywords (Figure 9D) and to create a timeline view (Figure 9E). The clusters obtained by the analysis were mainly “#0 enteric nervous system, #1 gut microbiota, #2 cardiolipin, #3 microbiota-gut-brain axis, and #4 tumor-associated. monocytes, #5 cd4(+) t cells, #6 central nervous system, #7 community.” The timeline view reflects the relationship between hot spots and time (Figure 9E). Nodes positioned toward the right represent more recent time points, whereas those on the left indicate older time periods. The latest axis on the timeline corresponds with our clustered labels, suggesting that the current research focus is on the relationship between ferroptosis in glioma and gut microbiota, enteric nerves, immune cells, and lipid metabolism.

**TABLE 3:** Top 20 keywords for ferroptosis in gliomas.

Rank	Keyword	Occurrences	Rank	Keyword	Occurrences
1	Ferroptosis	246	11	Oxidative stress	13
2	Glioblastoma	108	12	LncRNA	11
3	Glioma	87	13	Biomarker	10
4	Prognosis	37	14	GBM	10
5	Autophagy	26	15	Lipid peroxidation	10
6	Immunotherapy	22	16	Nrf2	10
7	Cell death	21	17	Temozolomide	10
8	Cancer	17	18	Tumor microenvironment	10
9	gpx4	16	19	Drug resistance	9
10	Apoptosis	14	20	Erastin	9

### 3.7 Thematic evolution and thematic maps

**FIGURE 10:**
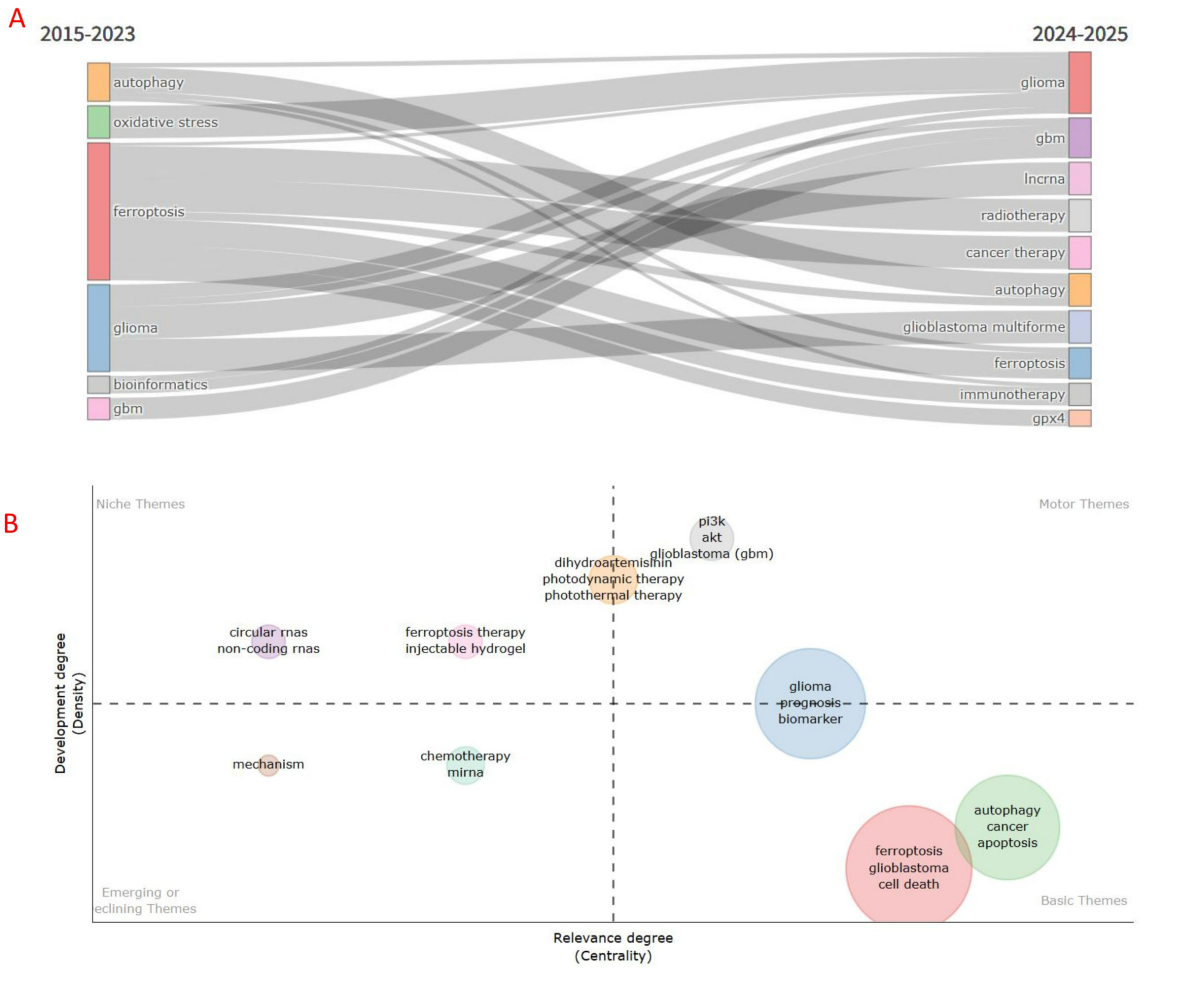
Keyword evolution based on two time periods **(A)** and thematic map based on keywords **(B)**.

In bibliometrics, it is essential to conduct co-occurrence and cluster analyses of keywords in the literature to understand the development and evolution of topics within the research field. The evolution of themes over time is visualized using the R package “bibliometrix.” In Figure 10A, we can see that the research topics of ferroptosis in gliomas from 2015 to 2023 are focused on “glioblastoma,” “autophagy,” “bioinformatics” and “oxidative stress.” The research topics in 2024–2025 will focus on “glioblastoma,” “incRNA,” “immunotherapy,” “gpx4,” and “radiotherapy.”

Thematic maps are utilized to illustrate the internal connections and strengths of various topics, highlighting their positions and developments within the field. In Figure 10B, centrality (represented on the horizontal axis) indicates the relevance of a topic in relation to other topics, with those exhibiting higher centrality demonstrating greater significance within that research domain. Density (represented on the vertical axis) reflects the strength of the relationships among the keywords associated with a given topic. A higher density signifies a clearer research concept and a more advanced state of research. “Ferroptosis therapy,” “injectable hydrogel,” “pl3k,” “akt,” “dihydroartemisinin,” “photothermal therapy,” and “photodynamic therapy” and other related topics are of great significance in the research of ferroptosis in gliomas and have been well developed. In addition, studies on ferroptosis-related “circular RNA” and “non-coding RNA” in gliomas have been mature. The new or declining themes were “mechanism,” “chemotherapy,” and “miRNA.”

## 4 Discussion

### 4.1 Information

In the past 11 years, the number of papers related to ferroptosis in gliomas has continuously increased, and research in related fields is consistently expanding. This trend indicates that the research strategy surrounding ferroptosis in gliomas is gradually maturing, attracting increasing attention from scholars. Over this period, researchers have developed a relatively comprehensive understanding of the mechanisms underlying ferroptosis. More researchers are increasingly exploring the potential relationship between glioma progression and ferroptosis. They are currently focused on inhibiting glioma progression and enhancing the effectiveness of chemotherapy by activating specific targets to induce ferroptosis in tumor cells. With the efforts of researchers, it has been discovered that ferroptosis, a mode of programmed cell death, plays multiple roles in glioma (Luo et al., 2022). Iron in the brain is involved in various vital activities of brain cells and plays a crucial role in maintaining the normal functioning of the central nervous system. In addition, it is noteworthy that iron transporter cells are mainly expressed in glial cells (Yu and Chang, 2019). Iron serves as a cofactor for numerous enzymes. Research has indicated that the concentration of free iron in gliomas is significantly higher than that found in normal tissues, particularly in glioblastomas (Park et al., 2019). TFR2 is a crucial gene involved in iron transport, and its expression in gliomas is significantly higher than in other tissues. Research has shown that the proliferation of gliomas is dependent on TFR2 (Hänninen et al., 2009). GPX4, ACSL4, P53, and FTH1 are currently recognized as biomarkers of ferroptosis in gliomas (Li et al., 2020; Yang et al., 2014); however, they are not considered the gold standard, and further auxiliary studies are necessary to validate their efficacy. In summary, existing studies primarily assess whether patients can benefit from ferroptosis based on three criteria: iron levels, gene expression, and mutations. Identifying significant biomarkers of ferroptosis remains a global challenge. Therefore, it is essential for researchers worldwide to collaborate in order to obtain the necessary indicators more quickly and effectively.

Through our analysis, we found that among the top ten countries in global publications, China and the United States are the primary leaders in ferroptosis research related to glioma. There has been extensive collaboration between these two countries; however, cooperation with other nations remains limited, and the intensity of such collaboration is insufficient. We urge as many countries as possible to engage in research on ferroptosis in glioma and to foster more in-depth communication. China and the United States are recognized authorities in this field. This trend may be attributed to China’s large population, which provides a greater number of case samples for analysis, as well as the advanced medical science, technology, and research equipment available in the United States, which facilitate more comprehensive investigations.

In our analysis of research institutions, we found that nearly all of the top 10 institutions in terms of publications were from China. The primary research institutions in this discipline include Nanjing Medical University, Central South University, Shandong University, and Southern Medical University. However, there is minimal collaboration between Chinese institutions and those in other countries, which may hinder the long-term progress of research in this field. Therefore, given the limited resources and conditions, we recommend that more extensive collaboration and interaction among institutions be pursued to enhance their competitiveness and scientific research output.

The analysis of academic journals enables us to identify the most influential publications within the discipline and the most appropriate journals for submission. Peer review is a crucial step in the publication of scholarly literature. Frontiers in Oncology has published the most research on this subject, with 27 articles released over the past 11 years and an impact factor (IF) of 3.5. Consequently, enhancing the global impact of journals is vital for the advancement of this discipline.

From the author’s analysis, Lu Shan, Wang Xuanzhong, Chen Qianxue, Sun Qian, and Xu Yang have published the most articles. Their research focuses on analyzing the regulatory mechanisms of key target genes involved in the induction of ferroptosis in glioma. A study published by Lu Shan and Wang Xuanzhong suggested that ATF3 could upregulate NOX4 and downregulate xCT and catalase, thereby promoting the accumulation of H2O2 in cells. Hydrogen peroxide (H2O2) can activate transferrin receptor (TFR) and lead to iron overload, which induces cell death in glioma (Lu et al., 2021). Xu Yang, Sun Qian, and Chen Qianxue’s collaborative study found that the activation of TFR2 can enhance the therapeutic efficacy of temozolomide in treating glioma (Tong et al., 2023). This discovery offers an effective adjuvant treatment strategy for improving the clinical prognosis of glioma patients (Tong et al., 2023). This discovery offers an effective adjuvant treatment strategy for improving the clinical prognosis of glioma patients. Their work represents a significant focus within this discipline. From these studies, we understand that researchers should concentrate on analyzing the regulatory mechanisms of key target genes involved in the induction of ferroptosis in glioma to enhance the efficacy of chemotherapy. In summary, their study offers a variety of induction targets for ferroptosis in glioma, enriches the understanding of the mechanisms of action, and provides practical and effective research programs and ideas for future researchers. However, despite existing collaborations, the partnerships among researchers remain insufficiently extensive and deep, which may hinder the advancement of this discipline. Therefore, we recommend that researchers organize more comprehensive academic conferences to facilitate communication within this field. In the analysis of co-cited authors, Dixon, SJ are the most frequently cited, and he is recognized as an authority in this field. Dixon, SJ elucidated the intricate cellular biological processes of ferroptosis, including the accumulation of lipid peroxidation and alterations in cell metabolism. This work serves as a foundational reference for many subsequent researchers conducting analyses and designing studies in this discipline.

Through an analysis of fundamental bibliometric data, we found that since 2020, research on ferroptosis in glioma has been steadily increasing both domestically and internationally, particularly among Chinese scholars. The scope of published articles includes numerous reputable international journals with wide distribution; however, most of them concentrate on cytological research. Therefore, future research directions should aim to expand the research scope, move beyond the limitations of cytology, and engage in further investigation and analysis using organs or individuals as subjects of study. From the perspective of the issuing institutions, the distribution of articles is uneven, with the majority originating from Chinese universities and a notable lack of collaboration with professional research institutes and foreign organizations. Furthermore, most major research institutions are concentrated in southern China, resulting in an uneven geographical distribution. The northern region of China must also generate significant enthusiasm for participation in relevant research. Additionally, during the research process, it is essential to conduct comprehensive data collection on glioma in collaboration with professional research institutes and to engage in international exchanges and cooperation. An analysis of the literature reveals that authors with a substantial number of published papers have concentrated their research on the action sites and mechanisms of ferroptosis in glioma. Therefore, in the future, alongside focusing on the mechanisms of ferroptosis in glioma, we should place greater emphasis on the translation of research findings. It is essential to conduct more in-depth analyses of the impact of this research on populations and individuals. Additionally, we must validate existing findings through animal experiments and clinical trials, with the goal of applying these insights to clinical practice as soon as possible to enhance the prognosis for glioma patients.

### 4.2 Research basis and research hotspots

Co-cited literature refers to works that are cited together by researchers. The research foundation of ferroptosis in glioma can be established through co-citation analysis. In this study, VOSviewer was utilized to categorize the literature into three distinct families, and a network map of co-cited open literature was created. Notably, the blue cluster is particularly prominent. The literature within the blue cluster primarily focuses on cell and animal experiments, elucidating the form and specific mechanisms of ferroptosis. Ferroptosis is a regulated form of cell death characterized by the iron-dependent accumulation of lipid hydroperoxides. Cancer cells are often vulnerable to disruptions in thiol metabolism, and excessive iron accumulation can exert its carcinogenic effects through oxidative stress (Toyokuni et al., 2017). In addition, a significant increase in reactive oxygen species (ROS) levels can be detected in cells undergoing ferroptosis due to the disruption of cysteine or glutathione metabolism (Liu et al., 2017). GPX4 is a crucial regulator in cancer cell research and ferroptosis. The increase in lipid peroxidation following GPX4 inhibition serves as the biological basis for ferroptosis in tumor cells (Seiler et al., 2008). At present, the commonly used ferroptosis inducers include the following four categories (Yang and Stockwell, 2016): (I) systemic XC-inhibitors (erastin and its analogs); (II) GPX4 inhibitors, such as RSL3; (III) depletes FIN56 of GPX4 protein and coQ10, a lipophilic antioxidant; (IV) FINO2, which indirectly inhibits GPX4 activity and activates the lipid peroxidation pathway. The green cluster focuses on analyzing key target genes and regulatory pathways of ferroptosis in cancer cells. Studies have demonstrated that FSP1 acts synergistically with GPX4 and glutathione to inhibit ferroptosis in cancer cells, thereby promoting cancer progression (Doll et al., 2019). In contrast, the activation of the ACSL4 and Nrf2-Keap1 pathways can induce ferroptosis in cancer cells, contributing to cancer suppression (Cheng et al., 2020; Fan et al., 2017). The red cluster is primarily focused on review analysis, emphasizing the application and research prospects of ferroptosis in cancer treatment. Studies have demonstrated that ferroptosis holds significant therapeutic potential for tumor suppression and immune surveillance, with many tumor suppressors exhibiting sensitivity to ferroptosis. P53 gene can promote the ferroptosis of tumor cells by inhibiting the transcription of the system XC- subunit SLC7A11 (Jiang et al., 2015). BAP1 induces ferroptosis in tumor cells by downregulating SLC7A11 expression (Zhang et al., 2018). Studies have also found that the combination of immunotherapy and ferroptosis inducers can enhance one another and produce synergistic effects (Wang et al., 2019), providing new insights into the treatment of glioma. In addition, natural molecules such as glutamic acid, p53, iron, radiation, and polyunsaturated fatty acid phospholipids (PUFA-PLs), which may induce iron ptosis, are expected to demonstrate significant research value in the future (Jiang et al., 2021).

The first comprehensive explanation of ferroptosis was presented in a study by Dixon et al. (2012). The cell models utilized in this research included RAS mutant cell lines (HT-1080, BJeLR, Calu-1). Reactive oxygen species (ROS) production was assessed using H2DCFDA, C11-BODIPY, and MitoSOX, while ATP levels were measured to differentiate between cell necrosis and ferroptosis. The involvement of iron and ROS was further confirmed through treatments with an iron chelator (DFO) and an antioxidant (trolox). At the genetic level, essential genes associated with ferroptosis, such as IREB2, ACSF2, and CS, were identified through shRNA library screening. Cellular functions were validated by siRNA knockdown or overexpression of key genes, including SLC7A11. After a comprehensive analysis, the researchers defined ferroptosis as a novel form of iron-dependent, lipid reactive oxygen species (ROS)-driven non-apoptotic cell death. This mechanism of cell death is fundamentally distinct from apoptosis, necrosis, and autophagy (Dixon et al., 2012). This study serves as the foundation for all research on ferroptosis and offers a robust theoretical framework for analyzing ferroptosis in glioma.

The second highest co-citation was the study published by Yang et al. (2014). In their research, they induced ferroptosis in tumor cells using erastin and RSL3, and analyzed the changes in polar and lipid metabolites in erastin-treated HT-1080 fibrosarcoma cells through LC-MS/MS technology. Their findings revealed glutathione (GSH) depletion and lysophosphatidylcholine (lysoPC) accumulation. The direct target protein of RSL3, GPX4, was identified using an affinity-labeled probe (RSL3-fluorescein) coupled with mass spectrometry. Finally, the regulatory role of GPX4 in ferroptosis was confirmed through overexpression and siRNA knockdown experiments. Lipid reactive oxygen species (ROS) were detected using BODIPY-C11, and the characteristics of ferroptosis were validated with an iron chelator (DFOM), a MEK inhibitor (U0126), and an antioxidant (vitamin E). After a comprehensive analysis, GPX4 was identified as a core regulatory protein of ferroptosis. Its inactivation led to the accumulation of lipid reactive oxygen species (ROS) and subsequent cell death (Yang et al., 2014). In addition, this study identified PTGS2, a downstream marker of ferroptosis that is upregulated during the occurrence of ferroptosis (Yang et al., 2014). This finding may be useful for evaluating the adjuvant therapeutic effects of ferroptosis in glioma in the future.

### 4.3 Hotspots and Frontiers

Keyword co-occurrence analysis can help us quickly identify the hotspots and Frontiers of this research. Through the frequency of keywords, we found that “prognosis,” “autophagy,” “gpx4,” “immunotherapy,” “oxidative stress,” “incRNA,” “biomarker,” “lipid peroxidation,” “NFR2,” “temozolomide treatment,” “tumor microenvironment” and other keywords appeared most frequently. These key words are closely related to the study of ferroptosis in gliomas. These keywords indicate that prior studies on ferroptosis in gliomas have concentrated on immune regulation and mechanisms of action. The results of trend subject headings analysis showed that “oxidative stress,” “NFR2,” and “copper mortality” were the focus of research after 2023. Through the thematic evolution, we found that “incRNA,” “immunotherapy,” “gpx4” and “radiotherapy” were the research priorities after 2024. Through comprehensive analysis, it was found that “gpx4” and “oxidative stress” may be the potential mechanism of action linking these studies. Then, through keyword clustering and a timeline view, we found that the latest research can be divided into eight modules: the enteric nervous system, gut microbiota, cardiolipin, microbiota-gut-brain axis, tumor-associated monocytes and CD4(+) T cells, central nervous system, and community. Yu et al.’s (2024) study highlights that ferroptosis has emerged as a crucial regulatory mechanism in diseases affecting the gut-liver-brain axis, primarily through oxidative stress (reactive oxygen species, or ROS, and lipid peroxidation) and the Nrf2/HO-1 signaling pathway. This discovery opens new avenues for the treatment of cross-organ diseases, such as targeting GPX4 or modulating intestinal flora (Yu et al., 2024). While this represents a promising direction for future research on the relationship between glioma and ferroptosis, challenges related to organ-specific markers and bidirectional regulatory mechanisms must be addressed moving forward. In addition, immune factors and immunotherapy will continue to be the focus of research in the coming years. In addition, immune factors and immunotherapy will continue to be the focus of research in the coming years. Studies have identified that ferroptosis inhibitors, such as CD44, HSPB1, and SLC40A1, are significantly associated with the prognosis of glioblastoma, potentially influencing outcomes through immunosuppression (Deng et al., 2021). However, the specific mechanisms of action remain unclear, and further research is necessary to confirm these findings. Through the thematic map, we discovered that research on the mechanisms of ferroptosis in glioma is relatively advanced, with some findings already being applied in clinical treatments. Future research should focus on translating more theoretical insights into clinical applications. “miRNA, chemotherapy” as emerging topics in this discipline also needs to be given more attention. Improving the chemotherapeutic efficacy against glioma by combining ferroptosis-inducing agents is a key focus of our future research.

In conclusion, ferroptosis as a novel cell death modality can significantly improve the prognosis of glioma. Investigating the regulatory mechanisms of ferroptosis in glioma is essential for enhancing our understanding of the onset, progression, and alterations in the immune microenvironment associated with this type of cancer, thereby providing new insights into its pathology. Furthermore, in contrast to conventional treatment modalities, targeting ferroptosis in tumor cells minimizes damage to healthy cells and holds significant practical application value (Hao et al., 2024). Current research on ferroptosis in glioma remains in its nascent stages, revealing a dual role of ferroptosis in this context, wherein it can both inhibit and promote cancer progression (Yeon Kim et al., 2024), and the specific regulatory mechanism is not yet perfect, and only cell and animal experiments, without clinical trials. Therefore, active exploration of ferroptosis in gliomas will lead to more groundbreaking research results in the future.

From the reference analysis, we found that some scholars employed a mixed academic language to describe the concept, nature, characteristics, mechanisms, targets, and other related aspects of ferroptosis. This has contributed to the complexity of the research content surrounding ferroptosis, rendering the investigation of its characteristics more diverse yet ambiguous. Therefore, it is essential to enhance academic rigor to ensure the scientific validity and authority of research findings. Keyword analysis indicates that current research levels are varied and perspectives are diverse. However, this also highlights the lack of a unified understanding of the characteristics of ferroptosis in gliomas. As research methods continue to evolve and improve, it is crucial to accurately identify the key characteristics and landmark molecules of ferroptosis, thereby providing a more precise theoretical foundation for the study of ferroptosis in gliomas.

### 4.4 The value and limitation of this study

Bibliometrics offers a novel avenue for exploring the research related to ferroptosis in gliomas. This approach enhances our understanding of its potential value in glioma treatment and provides new directions for clinical interventions. On the basis of bibliometrics, an in-depth exploration and application of various methods to induce ferroptosis in gliomas is anticipated to enhance the prognosis for glioma patients. Simultaneously, the preliminary bibliometric analysis offers a theoretical foundation for clinical practice and, to some extent, fosters the development of clinical drugs and the emergence of novel treatment methods. In addition, we used three different bibliometrics tools, among which VOSviewer and CiteSpace are widely used in bibliometrics analysis (Pan et al., 2018). The process of our analysis is relatively objective and reliable. However, this article has some limitations. This paper primarily employs bibliometric analysis to examine the English literature within the Web of Science Core Collection (WoSCC) database. Some significant studies published in other languages, such as Chinese, may have been overlooked. Additionally, due to technical limitations, our research did not encompass studies from other databases, such as PubMed, which may have resulted in the omission of important international articles. Even so, the studies included in the Web of Science Core Collection (WoSCC) database are extensive enough to allow us to trace the development and research focus of ferroptosis in gliomas over the past 20 years, based on the literature available in this database. This database was selected because it is our most frequently used bibliometric citation database, providing us with accurate and timely updates on the literature. Although there are some technical limitations in this study due to reliance on the Web of Science Core Collection (WoSCC) database, we can provide a preliminary summary of the research in this field and outline a blueprint for its future development. Following future technical updates, multiple literature databases can be integrated for a more comprehensive analysis.

## 5 Conclusion and prospect

Through bibliometric analysis, we found that the study of ferroptosis in glioma holds significant clinical application value and vast developmental prospects. The substantial increase in the number of publications since 2022 indicates that research in this field is entering a “golden age.” Furthermore, global interest in this area is growing year by year. To this end, it is essential to enhance cooperation and exchanges among countries and agencies. Currently, research on ferroptosis in glioma has yielded valuable results by focusing on therapeutic targets, molecular mechanisms, and immunotherapy. “GPX4” and “reactive oxygen species” are critical sites of action and manifestations of ferroptosis in glioma. The production of reactive oxygen species (ROS) in cells can be regulated by influencing the GPX4 axis pathway, thereby promoting the occurrence of ferroptosis (Banu et al., 2024). In addition, studies have demonstrated that GPX4 can regulate peroxidation-induced ferroptosis in certain developing brain cell types through sulfhydryl-independent selenate catalysis (Ingold et al., 2018). However, the role of GPX4’s independent ferroptosis regulatory mechanism in various states of glioma cells requires further investigation, which will be a primary focus of our future research. In recent years, immunotherapy has garnered significant attention in glioma research. The presence of a tumor-immunosuppressive environment is a primary factor contributing to the poor prognosis associated with glioma immunotherapy. Notably, as research has advanced, an increasing number of scholars have discovered that ferroptosis is closely linked to the progression of glioma. Targeted induction of ferroptosis in tumor cells may reshape the tumor immune microenvironment, potentially influencing the outcomes of immunotherapy (Chen et al., 2021). In summary, while ferroptosis demonstrates significant therapeutic potential in the treatment of glioma, several challenges remain. These challenges can be categorized as follows: I. The precise mechanisms underlying ferroptosis have yet to be fully elucidated, and there is currently no established “gold standard” for its assessment. The process is typically influenced by the interplay of multiple factors, such as GPX4 and p53 (Li et al., 2020; Yang et al., 2014). The convergence of these targets complicates research efforts for scholars. II. Ferroptosis exhibits a dual role in cancer, both inhibiting and promoting tumor growth (Wang et al., 2022). Further investigation is required to develop treatment strategies that effectively balance these opposing effects to enhance the prognosis for glioma patients. III. Following ferroptosis, a variety of signaling molecules are released (Xie et al., 2016), yet the functions and implications of these molecules remain largely unknown. IV. The relationship between ferroptosis and other modes of cell death is not well understood (Jiang et al., 2021). V. There is an interaction between ferroptosis and surrounding immune cells (Jiang et al., 2021), and the specific mechanisms of this interaction warrant further analysis in future studies. VI. The detailed mechanisms by which ferroptosis contributes to cellular immune tolerance are still unclear, and further research is needed to clarify how it promotes immune tolerance in glioma. Although many mechanisms of ferroptosis in glioma remain unclear, further research and technological advancements—such as single-cell technology and transcriptomics—are expected to gradually address these issues. Ferroptosis is anticipated to play a significant role in the treatment of glioma. Furthermore, the limited research findings available at this stage have been successfully integrated into clinical adjuvant therapy, yielding relatively positive outcomes. Therefore, in addition to conducting fundamental experiments, greater emphasis should be placed on translating experimental results into clinical applications as promptly as possible.

## Data Availability

Publicly available datasets were analyzed in this study. This data can be found here: https://webofscience.clarivate.cn/.

## Footnotes

1
https://www.webofscience.com/wos/woscc/basic-search

2
https://www.bibliometrix.org

## References

[B1] Agbo F. Oyelere S. Suhonen J. Tukiainen M. (2021). Scientific production and thematic breakthroughs in smart learning environments: A bibliometric analysis. *Smart Learn. Environ.* 8:1. 10.1186/s40561-020-00145-4 40477293 PMC7810194

[B2] Banu M. Dovas A. Argenziano M. Zhao W. Sperring C. Cuervo Grajal H. (2024). A cell state-specific metabolic vulnerability to GPX4-dependent ferroptosis in glioblastoma. *EMBO J.* 43 4492–4521. 10.1038/s44318-024-00176-4 39192032 PMC11480389

[B3] Bray F. Laversanne M. Sung H. Ferlay J. Siegel R. Soerjomataram I. (2024). Global cancer statistics 2022: Globocan estimates of incidence and mortality worldwide for 36 cancers in 185 countries. *CA Cancer J. Clin.* 74 229–263. 10.3322/caac.21834 38572751

[B4] Brika S. Algamdi A. Chergui K. Musa A. Zouaghi E. (2021). Quality of higher education: A bibliometric review study. *Front. Educ.* 6:666087. 10.3389/feduc.2021.666087 PMC8929398 35308075

[B5] Chen C. Song M. (2019). Visualizing a field of research: A methodology of systematic scientometric reviews. *PLoS One* 14:e0223994. 10.1371/journal.pone.0223994 31671124 PMC6822756

[B6] Chen Q. Wang W. Wu Z. Chen S. Chen X. Zhuang S. (2021). Over-expression of lncRNA TMEM161B-AS1 promotes the malignant biological behavior of glioma cells and the resistance to temozolomide via up-regulating the expression of multiple ferroptosis-related genes by sponging hsa-miR-27a-3p. *Cell Death Discov.* 7:311. 10.1038/s41420-021-00709-4 34689169 PMC8542043

[B7] Cheng J. Fan Y. Liu B. Zhou H. Wang J. Chen Q. (2020). ACSL4 suppresses glioma cells proliferation via activating ferroptosis. *Oncol. Rep.* 43 147–158. 10.3892/or.2019.7419 31789401 PMC6912066

[B8] Comba A. Faisal S. Varela M. Hollon T. Al-Holou W. Umemura Y. (2021). Uncovering spatiotemporal heterogeneity of high-grade gliomas: From disease biology to therapeutic implications. *Front. Oncol.* 11:703764. 10.3389/fonc.2021.703764 34422657 PMC8377724

[B9] Cruz-Cárdenas J. Zabelina E. Guadalupe-Lanas J. Palacio-Fierro A. Ramos-Galarza C. (2021). COVID-19, consumer behavior, technology, and society: A literature review and bibliometric analysis. *Technol. Forecast Soc. Change* 173:121179. 10.1016/j.techfore.2021.121179 34511647 PMC8418327

[B10] Deng S. Zheng Y. Mo Y. Xu X. Li Y. Zhang Y. (2021). Ferroptosis suppressive genes correlate with immunosuppression in glioblastoma. *World Neurosurg.* 152 e436–e448. 10.1016/j.wneu.2021.05.098 34062295

[B11] Dixon S. Lemberg K. Lamprecht M. Skouta R. Zaitsev E. Gleason C. (2012). Ferroptosis: An iron-dependent form of nonapoptotic cell death. *Cell* 149 1060–1072. 10.1016/j.cell.2012.03.042 22632970 PMC3367386

[B12] Doll S. Freitas F. Shah R. Aldrovandi M. da Silva M. Ingold I. (2019). FSP1 is a glutathione-independent ferroptosis suppressor. *Nature* 575 693–698. 10.1038/s41586-019-1707-0 31634899

[B13] Donthu N. Kumar S. Mukherjee D. Pandey S. Lim M. (2021). How to conduct a bibliometric analysis: An overview and guidelines. *J. Bus. Res.* 133 285–296. 10.1016/j.jbusres.2021.04.070

[B14] Fan Z. Wirth A. Chen D. Wruck C. Rauh M. Buchfelder M. (2017). Nrf2-Keap1 pathway promotes cell proliferation and diminishes ferroptosis. *Oncogenesis* 6:e371. 10.1038/oncsis.2017.65 28805788 PMC5608917

[B15] Fang X. Chen Z. Zhou W. Li T. Wang M. Gao Y. (2023). Boosting glioblastoma therapy with targeted pyroptosis induction. *Small* 19:e2207604. 10.1002/smll.202207604 37066699

[B16] Fang Y. Bai Z. Cao J. Zhang G. Li X. Li S. (2023). Low-intensity ultrasound combined with arsenic trioxide induced apoptosis of glioma via EGFR/AKT/mTOR. *Life Sci.* 332:122103. 10.1016/j.lfs.2023.122103 37730111

[B17] Ge X. Wang Z. Jiang R. Ren S. Wang W. Wu B. (2021). SCAMP4 is a novel prognostic marker and correlated with the tumor progression and immune infiltration in glioma. *Int. J. Biochem. Cell. Biol.* 139:106054. 10.1016/j.biocel.2021.106054 34390854

[B18] Hänninen M. Haapasalo J. Haapasalo H. Fleming R. Britton R. Bacon B. (2009). Expression of iron-related genes in human brain and brain tumors. *BMC Neurosci.* 10:36. 10.1186/1471-2202-10-36 19386095 PMC2679039

[B19] Hao W. Sun N. Fan Y. Chen M. Liu Q. Yang M. (2024). Targeted ferroptosis-immunotherapy synergy: Enhanced antiglioma efficacy with hybrid nanovesicles comprising NK cell-derived exosomes and RSL3-loaded liposomes. *ACS Appl. Mater. Interfaces* 16 28193–28208. 10.1021/acsami.4c04604 38776411 PMC11164066

[B20] Ingold I. Berndt C. Schmitt S. Doll S. Poschmann G. Buday K. (2018). Selenium utilization by GPX4 is required to prevent hydroperoxide-induced ferroptosis. *Cell* 172 409–422.e21. 10.1016/j.cell.2017.11.048 29290465

[B21] Jiang L. Kon N. Li T. Wang S. Su T. Hibshoosh H. (2015). Ferroptosis as a p53-mediated activity during tumour suppression. *Nature* 520 57–62. 10.1038/nature14344 25799988 PMC4455927

[B22] Jiang X. Stockwell B. Conrad M. (2021). Ferroptosis: Mechanisms, biology and role in disease. *Nat. Rev. Mol. Cell. Biol.* 22 266–282. 10.1038/s41580-020-00324-8 33495651 PMC8142022

[B23] Li T. Yang A. Liu G. Zou S. Chen Y. Ni B. (2021). Status quo and research trends of craniopharyngioma research: A 10-year bibliometric analyses (From 2011 to 2020). *Front Oncol.* 11:744308. 10.3389/fonc.2021.744308 34660308 PMC8516404

[B24] Li Y. Cao Y. Xiao J. Shang J. Tan Q. Ping F. (2020). Inhibitor of apoptosis-stimulating protein of p53 inhibits ferroptosis and alleviates intestinal ischemia/reperfusion-induced acute lung injury. *Cell Death Differ.* 27 2635–2650. 10.1038/s41418-020-0528-x 32203170 PMC7429834

[B25] Liu D. Duong C. Haupt S. Montgomery K. House C. Azar W. (2017). Inhibiting the system xC-/glutathione axis selectively targets cancers with mutant-p53 accumulation. *Nat. Commun.* 8:14844. 10.1038/ncomms14844 28348409 PMC5379068

[B26] Lu S. Wang X. He C. Wang L. Liang S. Wang C. (2021). ATF3 contributes to brucine-triggered glioma cell ferroptosis via promotion of hydrogen peroxide and iron. *Acta Pharmacol. Sin.* 42 1690–1702. 10.1038/s41401-021-00700-w 34112960 PMC8463534

[B27] Luo Y. Tian G. Fang X. Bai S. Yuan G. Pan Y. (2022). Ferroptosis and its potential role in glioma: From molecular mechanisms to therapeutic opportunities. *Antioxidants* 11:2123. 10.3390/antiox11112123 36358495 PMC9686959

[B28] Pan X. Yan E. Cui M. Hua H. (2018). Examining the usage, citation, and diffusion patterns of bibliometric mapping software: A comparative study of three tools. *J. Informetrics* 12 481–493. 10.1016/j.joi.2018.03.005

[B29] Park K. Kim J. Testoff T. Adams J. Poklar M. Zborowski M. (2019). Quantitative characterization of the regulation of iron metabolism in glioblastoma stem-like cells using magnetophoresis. *Biotechnol. Bioeng.* 116 1644–1655. 10.1002/bit.26973 30906984 PMC6693654

[B30] Peng X. Liu C. Li J. Bao Z. Huang T. Zeng L. (2023). A novel 25-ferroptosis-related gene signature for the prognosis of gliomas. *Front. Oncol.* 13:1128278. 10.3389/fonc.2023.1128278 37152018 PMC10157171

[B31] Puliyappadamba V. Hatanpaa K. Chakraborty S. Habib A. (2014). The role of NF-κB in the pathogenesis of glioma. *Mol. Cell. Oncol.* 1:e963478. 10.4161/23723548.2014.963478 27308348 PMC4905061

[B32] Racine J. S. (2012). RStudio: A platform-independent IDE for R and sweave. *J. Appl. Econ.* 27 167–172. 10.1002/jae.1278

[B33] Savita Verma N. (2020). A review study on big data analysis using R studio. *Int. J. Eng. Technol. Manag. Res.* 6 129–136. 10.29121/ijetmr.v6.i6.2019.402

[B34] Seiler A. Schneider M. Förster H. Roth S. Wirth E. Culmsee C. (2008). Glutathione peroxidase 4 senses and translates oxidative stress into 12/15-lipoxygenase dependent- and AIF-mediated cell death. *Cell. Metab.* 8 237–248. 10.1016/j.cmet.2008.07.005 18762024

[B35] Sidhu A. K. Sighn H. Virdi S. Kumar R. (2020). A bibliometric analysis on job stress using visualizing network. *J. Content Commun. Commun.* 12 21–29. 10.18332/ejm/115983 33537606 PMC7839164

[B36] Synnestvedt M. Chen C. Holmes J. (2005). CiteSpace II: Visualization and knowledge discovery in bibliographic databases. *AMIA Annu. Symp. Proc.* 2005 724–728. 16779135 PMC1560567

[B37] Thomas B. Joseph J. Jose J. (2023). Explorative bibliometric study of medical image analysis: Unveiling trends and advancements. *Sci. Vis.* 15 35–49. 10.26583/sv.15.5.04

[B38] Tong S. Hong Y. Xu Y. Sun Q. Ye L. Cai J. (2023). TFR2 regulates ferroptosis and enhances temozolomide chemo-sensitization in gliomas. *Exp. Cell. Res.* 424:113474. 10.1016/j.yexcr.2023.113474 36702193

[B39] Toyokuni S. Ito F. Yamashita K. Okazaki Y. Akatsuka S. (2017). Iron and thiol redox signaling in cancer: An exquisite balance to escape ferroptosis. *Free Radic. Biol. Med.* 108 610–626. 10.1016/j.freeradbiomed.2017.04.024 28433662

[B40] Upadhyayula P. Higgins D. Mela A. Banu M. Dovas A. Zandkarimi F. (2023). Dietary restriction of cysteine and methionine sensitizes gliomas to ferroptosis and induces alterations in energetic metabolism. *Nat. Commun.* 14:1187. 10.1038/s41467-023-36630-w 36864031 PMC9981683

[B41] van Eck N. Waltman L. (2010). Software survey: Vosviewer, a computer program for bibliometric mapping. *Scientometrics* 84 523–538. 10.1007/s11192-009-0146-3 20585380 PMC2883932

[B42] Wang K. Wang J. Zhang J. Zhang A. Liu Y. Zhou J. (2022). Ferroptosis in glioma immune microenvironment: Opportunity and challenge. *Front. Oncol.* 12:917634. 10.3389/fonc.2022.917634 35832539 PMC9273259

[B43] Wang W. Green M. Choi J. Gijón M. Kennedy P. Johnson J. (2019). CD8+ T cells regulate tumour ferroptosis during cancer immunotherapy. *Nature* 569 270–274. 10.1038/s41586-019-1170-y 31043744 PMC6533917

[B44] Xie Y. Hou W. Song X. Yu Y. Huang J. Sun X. (2016). Ferroptosis: Process and function. *Cell Death Differ.* 23 369–379. 10.1038/cdd.2015.158 26794443 PMC5072448

[B45] Yang W. Stockwell B. (2016). Ferroptosis: Death by lipid peroxidation. *Trends Cell. Biol.* 26 165–176. 10.1016/j.tcb.2015.10.014 26653790 PMC4764384

[B46] Yang W. SriRamaratnam R. Welsch M. Shimada K. Skouta R. Viswanathan V. (2014). Regulation of ferroptotic cancer cell death by GPX4. *Cell* 156 317–331. 10.1016/j.cell.2013.12.010 24439385 PMC4076414

[B47] Yeon Kim S. Tang M. Lu T. Chih S. Li W. (2024). Ferroptosis in glioma therapy: Advancements in sensitizing strategies and the complex tumor-promoting roles. *Brain Res.* 1840:149045. 10.1016/j.brainres.2024.149045 38821335 PMC11323215

[B48] Yu P. Chang Y. (2019). Brain iron metabolism and regulation. *Adv. Exp. Med. Biol.* 1173 33–44. 10.1007/978-981-13-9589-5_3 31456204

[B49] Yu X. Wang S. Ji Z. Meng J. Mou Y. Wu X. (2024). Ferroptosis: An important mechanism of disease mediated by the gut-liver-brain axis. *Life Sci.* 347:122650. 10.1016/j.lfs.2024.122650 38631669

[B50] Zhang Y. Shi J. Liu X. Feng L. Gong Z. Koppula P. (2018). BAP1 links metabolic regulation of ferroptosis to tumour suppression. *Nat. Cell. Biol.* 20 1181–1192. 10.1038/s41556-018-0178-0 30202049 PMC6170713

